# Dexmedetomidine versus standard care sedation with propofol or midazolam in intensive care: an economic evaluation

**DOI:** 10.1186/s13054-015-0787-y

**Published:** 2015-02-19

**Authors:** Heidi Turunen, Stephan M Jakob, Esko Ruokonen, Kirsi-Maija Kaukonen, Toni Sarapohja, Marjo Apajasalo, Jukka Takala

**Affiliations:** Orion Corporation Orion Pharma, Orionintie 1, FI-02100 Espoo, Finland; Department of Intensive Care Medicine, Bern University Hospital and University of Bern, Freiburgstrasse, CH-3010 Bern, Switzerland; Department of Intensive Care Medicine, Kuopio University Hospital, Puijonlaaksontie 2, FI-70210 Kuopio, Finland; Australian and New Zealand Intensive Care Research Centre, Department of Epidemiology and Preventive Medicine, Monash University, 99 Commercial Road, 3004 Melbourne, Victoria Australia; Intensive Care Units, Helsinki University Central Hospital, Haartmaninkatu 2, 00029 HUS Helsinki, Finland

## Abstract

**Introduction:**

Dexmedetomidine was shown in two European randomized double-blind double-dummy trials (PRODEX and MIDEX) to be non-inferior to propofol and midazolam in maintaining target sedation levels in mechanically ventilated intensive care unit (ICU) patients. Additionally, dexmedetomidine shortened the time to extubation versus both standard sedatives, suggesting that it may reduce ICU resource needs and thus lower ICU costs. Considering resource utilization data from these two trials, we performed a secondary, cost-minimization analysis assessing the economics of dexmedetomidine versus standard care sedation.

**Methods:**

The total ICU costs associated with each study sedative were calculated on the basis of total study sedative consumption and the number of days patients remained intubated, required non-invasive ventilation, or required ICU care without mechanical ventilation. The daily unit costs for these three consecutive ICU periods were set to decline toward discharge, reflecting the observed reduction in mean daily Therapeutic Intervention Scoring System (TISS) points between the periods. A number of additional sensitivity analyses were performed, including one in which the total ICU costs were based on the cumulative sum of daily TISS points over the ICU period, and two further scenarios, with declining direct variable daily costs only.

**Results:**

Based on pooled data from both trials, sedation with dexmedetomidine resulted in lower total ICU costs than using the standard sedatives, with a difference of €2,656 in the median (interquartile range) total ICU costs—€11,864 (€7,070 to €23,457) versus €14,520 (€7,871 to €26,254)—and €1,649 in the mean total ICU costs. The median (mean) total ICU costs with dexmedetomidine compared with those of propofol or midazolam were €1,292 (€747) and €3,573 (€2,536) lower, respectively. The result was robust, indicating lower costs with dexmedetomidine in all sensitivity analyses, including those in which only direct variable ICU costs were considered. The likelihood of dexmedetomidine resulting in lower total ICU costs compared with pooled standard care was 91.0% (72.4% versus propofol and 98.0% versus midazolam).

**Conclusions:**

From an economic point of view, dexmedetomidine appears to be a preferable option compared with standard sedatives for providing light to moderate ICU sedation exceeding 24 hours. The savings potential results primarily from shorter time to extubation.

**Trial registration:**

ClinicalTrials.gov NCT00479661 (PRODEX), NCT00481312 (MIDEX).

**Electronic supplementary material:**

The online version of this article (doi:10.1186/s13054-015-0787-y) contains supplementary material, which is available to authorized users.

## Introduction

Intensive care unit (ICU) costs can take up to 20% of hospital budgets [1]. The daily costs of mechanically ventilated patients may be 20% to 44% higher than those of non-ventilated patients [2-5]. Therefore, shortening the time to extubation and the duration of mechanical ventilation (MV) are among key factors in reducing total ICU resource utilization and the respective ICU costs.

Mechanically ventilated ICU patients are commonly sedated to facilitate tolerance to artificial airway and other interventions. Dexmedetomidine, a highly selective alfa-2-agonist, was approved in Europe in 2011 for light to moderate ICU sedation. Recent studies suggest that dexmedetomidine may reduce the time to extubation compared with standard sedation [6-8]. However, reductions in MV or length of stay in the ICU do not necessarily always lead to cost savings [9-11]. Therefore, using a cost-minimization approach [12], we aimed to evaluate the net effect of dexmedetomidine on total ICU costs and to compare it to sedation with propofol or midazolam (or per cohort, a 1:1 mix of both, annually) in ICU patients requiring prolonged MV in Europe.

## Methods

### Background and design of PRODEX and MIDEX

This is a secondary analysis based on resource utilization and daily ICU cost data from two randomized, double-blind, double-dummy trials of dexmedetomidine versus standard sedation (midazolam in MIDEX and propofol in PRODEX [8]) conducted in nine European countries. In these two trials, mechanically ventilated adult patients estimated to require sedation for at least a further 24 hours were randomly assigned in regard to study sedative within 72 hours from ICU admission. Sedation was monitored at least every 4 hours, and the drug infusion rate was adjusted to achieve and maintain the targeted light to moderate sedation levels; daily sedation stops and spontaneous breathing trials were included. Time to extubation was recorded. Duration of MV was calculated from randomization until patients remained free of any form of MV for 48 hours. Patients were followed until ICU discharge or death or for a maximum of 45 days. According to the protocol, this included those patients who discontinued study medication prematurely for any reason. The study protocols were approved by the ethics committees of the study centers/countries (Additional file 1). Written informed consent was obtained from the patient’s family, a legal representative, or both.

### Summary of the economic analysis

In terms of maintaining patients at the target sedation levels, the performance of dexmedetomidine was shown in these trials to be non-inferior versus the standard sedatives, with no difference in mortality [8]. Therefore, we chose to conduct a cost-minimization analysis [12], focusing purely on the net impact of dexmedetomidine on the costs of care.

The total ICU costs associated with each study sedative were calculated on the basis of the total dose of the study sedative consumed and the number of days each patient remained intubated, required non-invasive ventilation (NIV), or required ICU care without MV. The daily unit costs for the three consecutive ICU periods were set to decline toward discharge [9-11] on the basis of the observed reduction in mean daily TISS (Therapeutic Intervention Scoring System) points [13-15] between the periods. The resulting mean and median total ICU costs were summarized per study sedative and compared between groups. A base case analysis is presented, followed by a number of sensitivity analyses to validate the method.

### Resource utilization parameters

First, for each patient with informed consent who had received any study drug, we extracted the available ICU resource utilization parameters from the original trial data. These included the following: time to extubation, duration of MV (including non-invasive MV), duration of ICU stay, duration of total hospital stay, total consumption of the assigned study sedative, and the daily number as well as the cumulative sum of TISS points [13-15] during the ICU stay. All of the above time periods were defined to start from the point of randomization to study drug.

We reflected the actual resources consumed as follows: if there was no record of the respective events having commenced already, extubation or the end of MV or ICU discharge or a combination of these was considered to have taken place at the time of death or the move to palliative care. Thus, compared with the original analysis [8], the use of ‘worst ranking imputation’ was minimized and applied only on the time-related end points of survivors lost to follow-up or withdrawn from the study, if the event had not yet taken place before that. This included, for example, transferred patients, some of the patients who had prematurely discontinued study medication and for whom the relevant time points had not been captured, and a few patients who withdrew from the entire trial before having reached the relevant time points. ‘Worst ranking imputation’ implies that, whenever a time-related end point was missing, it was assumed that the respective event (for example, extubation/end of MV/ICU discharge, usually following one another in this order) had taken place at the next, actually recorded time point or, if no such date was available, at 45 days.

When economic assessment of ICU care is performed, it is inappropriate to use a single average daily ICU cost for the entire stay. Instead, the declining cost of ICU days over time needs to be taken into consideration to quantify the actual costs for the patients [9-11]. To enable this within our cost analysis, we further derived from the above resource parameters three consecutive ICU periods differing by ventilator use:

1. Time on invasive MV: (same as Time to extubation in original trial data)
2. Time on NIV: (derived as Duration of MV-Time to extubation)
3. Time off MV: (derived as Time from randomization to actual ICU discharge-Duration of MV).

### Unit cost assumptions

All trial sites (n = 78) were approached to define the applicable unit costs. Eventually, mean daily ICU costs (total operative costs, including staff wages from the past year, per bed day) were received from 18 trial centers, resulting in a mean cost of €1,702 per ICU day. Specific unit cost assumptions for each of these three ICU periods were further set through adjusting the mean daily ICU cost of the trial centers with TISS points—a measure of resource utilization and nursing workload in intensive care [13-15]. The daily TISS score typically decreases from admission to discharge, reflecting the diminishing need for ICU resources and interventions over time, as the patient’s condition improves. A copy of the scoring tool is available in Additional file 2.

In our pooled data of nearly 1,000 patients, the mean differences in average daily TISS points were +33% for days on invasive MV relative to days off MV and +26% for days on NIV versus days off MV, indicating a decrease in resource needs as the need for MV is reduced toward discharge. We therefore adjusted the mean daily ICU cost of the 18 centers (€1,702 per day) for the three ICU periods by using these observed relative differences. The lowest unit cost, that for the off MV days (that also are the last ICU days), was obtained through an iterative process, in which the three period-specific unit costs were set to such levels that the total ICU costs for the entire pooled trial cohort remained the same whether calculated with the single mean daily ICU cost of €1,702 or with the three period-specific unit costs. Thus, the final base case unit cost assumptions for the present analysis were the following:

- Time on invasive MV: €1,850 per day (+33% versus Day off MV)
- Time on NIV: €1,750 per day (+26% versus Day off MV)
- Time off MV: €1,390 per day.

Sedative acquisition costs were calculated by multiplying the total study sedative dose consumed per patient with the following unit costs (source: IMS Health, Danbury, CT, USA): €0.012 per 1 mg propofol (equals €12 per 1,000 mg vial), €0.160 per 1 mg midazolam (€0.8 per 5 mg ampoule), and €0.090 per 1 μg dexmedetomidine (€18 per 200 μg ampoule of *dexdor*®; source: Orion Pharma, Espoo, Finland).

### Statistical methods

The data on resource utilization were analyzed by using the non-parametric time-to-event (Gehan-Wilcoxon) test for time to extubation, duration of MV, and actual ICU stay until discharge. The Wilcoxon-Mann-Whitney test was used for the cumulative sum of TISS points.

Costs were calculated on an individual patient basis. Both mean (standard deviation) and median (interquartile range) total ICU costs are given per treatment group throughout the article. Unless otherwise indicated for a specific sensitivity analysis, the total ICU costs reported in the results include the study sedative acquisition costs. The distribution of the total ICU costs is truncated and positively skewed. Therefore, we applied the non-parametric bootstrapping approach [16] to assess the likelihood of dexmedetomidine resulting in lower total ICU costs versus the standard sedatives. In bootstrapping, the patient-level total ICU cost for each study treatment group is resampled from the original trial data 10,000 times (n = 1,000 per sample) and the difference in the total ICU costs is calculated for each iteration. All analyses were performed by using SAS® 9.2 for Windows (SAS Institute Inc., Cary, NC, USA).

### Sensitivity analyses

To explore the robustness of the results, several one-way sensitivity analyses were performed in the 1:1 pooled data testing the impact of using more conservative unit cost assumptions:

- (S1) Cost of standard sedatives set to zero
- (S2) Cost of dexmedetomidine increased to €22 per 200 μg (that is, €0.11 per 1 μg), representing the current maximum dexdor® price for hospitals in EU countries. The cost of providing NIV may vary depending on how such care is organized in practice. Therefore, we performed a sensitivity analysis assuming a lower and a higher unit cost for an ICU day on NIV:
- (S3) Cost of ‘Time on NIV’ decreased to €1,390 per 24 hours (equals 24-hour cost in ICU off MV)
- (S4) Cost of ‘Time on NIV’ increased to €1,850 per 24 hours (equals 24-hour cost in ICU on invasive MV).

We also conducted cost analyses:

- (S5) including only the population with observed data available from all three ICU periods, in order to explore the impact of censoring at time of death and the imputations made on missing data
- (S6) including only the patients of those 18 centers which provided their ICU unit cost information for this analysis.
- (S7a-b) To further validate the main costing method in our analysis, we used an alternative costing approach based on the cumulative sum of daily TISS points, so that the decrease in resource needs, and thus indirectly in the unit cost for each consecutive ICU day [9,10], is taken into consideration on a day-by-day basis rather than in three steps. In this analysis, the total cumulative sum of the daily TISS points during the ICU stay was multiplied by the unit cost of one TISS point, adding sedative acquisition costs on top, to obtain the ‘Total TISS-based ICU cost’ for each patient. First (S7a), a unit cost of €40 per TISS point was assumed for the analysis on the basis of the mean value of the available information from 13 of the 18 PRODEX/MIDEX study centers, which had provided also their daily ICU cost information. The cost per TISS point was available from fewer PRODEX/MIDEX study centers and fewer countries than the cost per ICU day used in the main costing method. Furthermore, the costs of the four Estonian ICUs, among the 13 centers from three countries where the local cost per TISS point was available, lowered the mean TISS point cost disproportionally. Therefore, in the second part of this analysis (S7b), we used an alternative unit cost of €50 per point, corresponding to the three daily ICU period-specific unit cost assumptions used in the base case analysis (see ‘Unit cost assumptions’ section above).
- (S8a-b) We explored two additional scenarios that consider the declining direct variable daily ICU costs only [9-11], assuming that they represent either 25% [17,18] or 50% [18] of the total ICU costs, as indicated by published European studies.
- (S9) Finally, to reflect the net cost implications of dexmedetomidine use in hospitals where the underlying daily ICU costs are either much lower or higher than in our base case, we also performed an analysis in which the three period-specific mean daily ICU unit costs were decreased or increased to match mean costs between €500 and €3,100 per any ICU day. This reflects a cost range somewhat wider than found in the literature (that is, €700 to €3,000 per day) [5,18-25]. Since the prices of the standard sedatives can vary between hospitals, to make the analysis more conservative toward dexmedetomidine, the sedative acquisition costs were set to zero for the standard sedatives but not for dexmedetomidine.

## Results

### Population

The pooled base case analysis consisted of 990 patients: 493 on dexmedetomidine and 497 on standard care. The respective figures were 247/250 in MIDEX and 246/247 in PRODEX. For the majority of the patients, the main reason for ICU admission was either medical or surgical (70% and 22% in MIDEX and 55% and 30% in PRODEX, respectively). Baseline demographics are similar to those of the intention-to-treat population [8], as only eight (0.8% of all) untreated or non-consenting patients were excluded (see Additional file 3: Table S1 for further details on handling missing data).

### Resource utilization

In this secondary economic analysis focusing on actual costs of care, dexmedetomidine shortened the time to extubation (*P* = 0.0003), the duration of total MV (*P* = 0.0052), and the duration of actual ICU stay (*P* = 0.0210) compared with pooled standard sedation (Table 1). No difference in hospital ward stay after ICU discharge was observed. For further details, see Additional file 4: Table S2.

**Table 1:** **Resource utilization**

**1a.**	**Time to extubation, hours**				***P*** **value**
	**Mean (SD)**		**Median (IQR)**		
	**Dexmedetomidine**	**Standard care**	**Dexmedetomidine**	**Standard care**	
Pooled	166.1 (219.5)	195.1 (220.6)	89.0 (46.0-188.0)	118.0 (53.0-231.0)	0.0003
MIDEX	183.8 (226.4)	214.9 (229.3)	95.0 (59.0-212.0)	143.0 (75.0-232.0)	0.0013
PRODEX	148.3 (211.3)	175.1 (210.0)	69.0 (39.0-164.0)	91.0 (44.0-231.0)	0.0405
**1b.**	**Duration of mechanical ventilation, hours**				***P*** **value**
	**Mean (SD)**		**Median (IQR)**		
	**Dexmedetomidine**	**Standard care**	**Dexmedetomidine**	**Standard care**	
Pooled	192.7 (241.1)	213.2 (229.1)	103.0 (51.0-215.0)	139.0 (70.0-265.0)	0.0052
MIDEX	199.2 (237.5)	231.4 (239.9)	115.0 (66.0-216.0)	146.5 (84.0-260.0)	0.0047
PRODEX	186.3 (245.0)	194.7 (216.7)	94.0 (45.0-211.0)	114.0 (47.0-266.0)	0.2049
**1c.**	**Duration of actual ICU stay, hours**				***P*** **value**
	**Mean (SD)**		**Median (IQR)**		
	**Dexmedetomidine**	**Standard care**	**Dexmedetomidine**	**Standard care**	
Pooled	271.3 (267.2)	296.0 (265.6)	166.5 (98.5-315.0)	196.0 (116.0-361.0)	0.0210
MIDEX	281.8 (265.0)	318.4 (265.8)	179.5 (115.0-341.0)	217.0 (126.0-365.0)	0.0131
PRODEX	260.8 (269.6)	273.3 (265.0)	146.0 (94.0-293.0)	169.0 (93.0-355.0)	0.4057
**1d.**	**Cumulative sum of TISS points**				***P*** **value**
	**Mean (SD)**		**Median (IQR)**		
	**Dexmedetomidine**	**Standard care**	**Dexmedetomidine**	**Standard care**	
Pooled	360 (375)	403 (360)	239 (152-426)	279 (177-503)	0.0030
MIDEX	353 (328)	419 (372)	239 (157-428)	300 (181-507)	0.0094
PRODEX	368 (419)	386 (347)	242 (139-421)	273 (170-501)	0.1064

Duration of ventilator use (1a-b), intensive care unit (ICU) stay (1c), and cumulative sum of Therapeutic Intervention Scoring System (TISS) points (1d) are derived from MIDEX and PRODEX for the economic analysis. In MIDEX, the standard care sedative was midazolam; in PRODEX, it was propofol. IQR, interquartile range (first to third); SD, standard deviation.

### ICU costs-base case analysis

**Figure 1:**
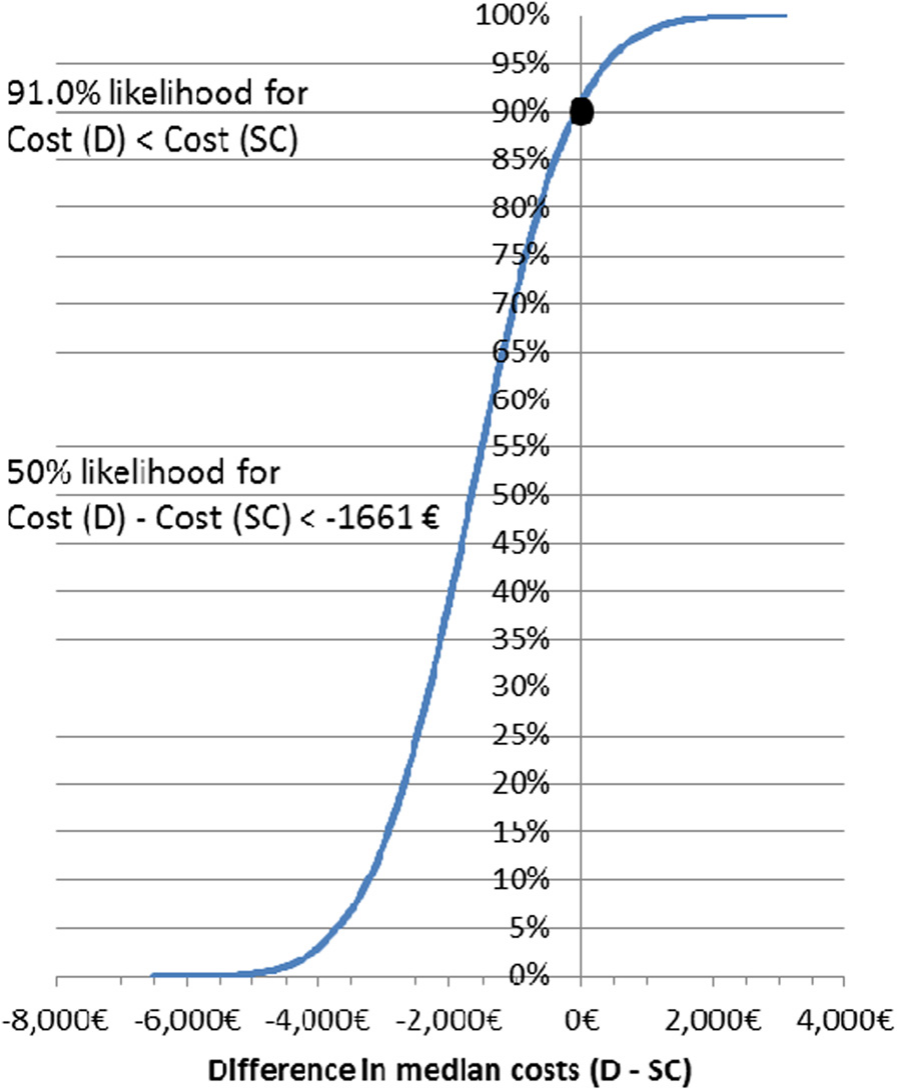
**The likelihood of dexmedetomidine (D) to result in lower total intensive care unit costs than pooled standard care sedatives (SC), assessed by bootstrapping.** As indicated by the black circle, the likelihood that dexmedetomidine results in lower total intensive care unit costs than the standard sedatives is 91.0%.

Median total ICU costs were €2,656 lower with dexmedetomidine versus pooled standard care sedatives (Table 2). The median costs with dexmedetomidine were €3,573 lower than midazolam and €1,292 lower than propofol despite higher sedative acquisition costs (see Additional file 5: Table S3 for sedative acquisition costs). Given the bootstrapping analysis of the 1:1 pooled data, dexmedetomidine is likely to lead to lower total ICU costs than the standard sedatives with a probability of 91.0% (Figure 1). The same assessment conducted separately from the PRODEX and MIDEX data sets indicated that, compared with propofol, dexmedetomidine is likely to decrease the total ICU costs with a probability of 72.4% but that the respective likelihood in comparison with midazolam is 98.1%. For further details, see Additional file 6: Figure S1.

**Table 2:** **Total intensive care unit costs with dexmedetomidine and current standard care sedatives, expressed as euros per patient**

	**Mean (SD)**		**Difference in means, €**	**Median (IQR)**		**Difference in medians, €**
	**Dexmedetomidine**	**Standard care**		**Dexmedetomidine**	**Standard care**	
Pooled	19,609 (19,568)	21,258 (19,298)	−1,649	11,864 (7,070-23,457)	14,520 (7,871-26,254)	−2,656
MIDEX	20,342 (19,412)	22,878 (19,483)	−2,536	12,871(8,126-25,147)	16,444 (8,955-27,676)	−3,573
PRODEX	18,872 (19,735)	19,619 (19,008)	−747	11,016 (6,781-21,696)	12,308 (6,787-25,247)	−1,292

In MIDEX, the standard care sedative was midazolam; in PRODEX, it was propofol. IQR, interquartile range; SD, standard deviation.

### ICU costs-sensitivity analyses

Table 3 shows the results of the sensitivity analyses S1-S8 in the pooled data. The difference in the total ICU costs observed in the base case analysis appeared to change only little when the assumptions on sedative unit costs and cost structures (the unit cost of NIV days) were varied. All variations in ICU unit costing (S1-S4) resulted in at least €2,515 lower costs with dexmedetomidine on the basis of median costs of each treatment group.

**Table 3:** **Sensitivity analyses S1-S8 in pooled data**

**Sensitivity analysis of the pooled data: dexmedetomidine versus standard care sedatives**	**Difference in mean costs, €**	**Difference in median costs, €**
S1. Standard sedative cost set to zero	−1,545	−2,515
S2. Dexmedetomidine cost increased to €22 per 200 μg (€0.11 per 1 μg)	−1,580	−2,591
S3. Cost of a NIV day (24 hours) decreased to €1,390	−1,779	−2,717
S4. Cost of a NIV day (24 hours) increased to €1,850	−1,614	−2,551
S5. Patients with observed data from all three time periods (no censoring, no imputation). N (dexmedetomidine): 366; N (standard care): 391	−3,213	−2,462
S6. Patients of those 18 study centers, from which the ICU unit costs were obtained. N (dexmedetomidine): 170; N (standard care): 166	−1,763	−4,567
S7a. TISS-based total ICU costs, at unit cost of €40 per TISS point	−1,499	−1,448
S7b. TISS-based total ICU costs, at unit cost of €50 per TISS point	−1,926	−1,782
S8a. Only declining direct variable daily costs included, assuming they represent 25% of total ICU costs	−256	−515
S8b. Only declining direct variable daily costs included, assuming they represent 50% of total ICU costs	−720	−1,343

The impact of using alternative assumptions for the unit costs was tested. Additionally, cost difference between treatment groups was evaluated in two subpopulations and through applying a different type of costing method based on the mean cumulative sum of daily TISS (Therapeutic Intervention Scoring System) points throughout the entire intensive care unit (ICU) stay with two different unit costs. Finally, two analyses including only direct variable costs are presented (8a-b). Further details of analyses S5-S8 can be found in Additional files 7, 8, and 9: Tables S4-8. NIV, non-invasive ventilation.

Sedation with dexmedetomidine resulted in lower ICU costs in pooled population also when considering only the patients (n = 757) with observed data available from all three time periods (S5; with no imputations or censoring) or only data (n = 336) from those study centers from which the underlying unit costs were received (S6: further information in Additional file 7: Tables S4 and S5).

The complementary cost analysis based on TISS points (S7, Table 3) indicated a difference of €1,448 (at €40 per point) and €1,782 (at €50 per point, S7b) in median ICU costs, favoring dexmedetomidine over pooled standard care (details in Additional file 8: Table S6).

When including only the declining, direct variable daily costs in the cost comparison (S8a-b), dexmedetomidine resulted in lower median costs than pooled standard care, the difference being €515 when variable costs were assumed to represent 25% of the total costs or €1,343 when assumed to be 50% (Table 3). Also, the sensitivity analyses comparing dexmedetomidine separately with propofol or midazolam indicated that dexmedetomidine sedation results in lower ICU costs (Additional file 9: Tables S7 and S8)*.*

**Figure 2:**
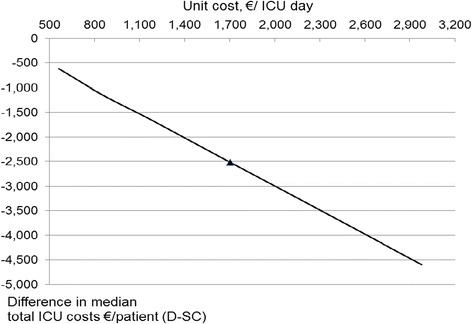
**Impact of the underlying mean intensive care unit (ICU) daily cost level on the total ICU cost difference between dexmedetomidine (D) and standard care (SC).** The three ICU period-specific daily costs were varied up or down from the base case values that reflected an overall mean cost of €1,702 per ICU day (triangle). Alternative period-specific unit costs reflecting a mean cost range between €500 and €3,100 per ICU day were tested. Negative values indicate lower costs on dexmedetomidine. For a conservative approach, the standard sedative acquisition costs were set to zero.

A comparison of the total ICU costs of dexmedetomidine and pooled standard care with different levels of period-specific daily ICU costs (S9, Figure 2) indicated that, even if the underlying mean daily ICU cost were as low as €500, dexmedetomidine sedation would still result in lower total ICU costs than using the standard sedatives. Naturally, if the unit costs are higher than in the base case, the cost benefit of dexmedetomidine increases.

## Discussion

This economic analysis suggests that the higher acquisition costs of dexmedetomidine compared with standard sedation may be compensated for by reductions in other ICU costs. In this study, dexmedetomidine resulted in lower ICU costs compared with standard care, primarily by reducing the time to extubation. In economic evaluations of ICU care, however, it is important to consider the net impact on the total duration of ICU and hospital stay [26]. In our analysis, dexmedetomidine reduced the duration of total ICU stay compared with standard care, without prolonging post-ICU ward stay. The finding of reduced time to extubation is supported by the results of the sensitivity analyses of the original studies [8] as well as similar findings in previous studies [6,7,27].

The estimated unit costs and the assumption of less expensive care after MV and ICU discharge are the key drivers for the potential cost savings from dexmedetomidine use. The unit cost assumptions were built on the mean daily ICU costs obtained from 18 trial centers, representing six out of nine study countries, and 34% of all patients included in this economic analysis. However, the intention of this study was not to calculate the precise costs for the specific ICUs who were involved in the trials but rather to provide a more general picture of the potential cost implications in different European settings, where daily ICU costs may vary quite substantially by country and center. In fact, the mean daily ICU cost of the 18 study centers appears to be well in line with the available cost references in the literature, indicating an approximate ICU cost range of €700 to €3,000 per day across Europe [5,18-25], a range that was well covered in the respective sensitivity analysis.

When an economic assessment of ICU care is performed, a single mean daily ICU cost should not be used; instead, the declining cost of ICU days over time needs to be taken into consideration [9-11]. In the present analysis, this was done in two different ways: first, using three decreasing unit cost levels tied to the decrease in MV use over time (base case analysis), and second, on a day-by-day basis, directly based on the daily TISS points of each patient, thus reflecting marginal costs (sensitivity analysis) [9-11]. Both of these costing approaches were driven by the daily decline in variable costs toward discharge and indicated similar results in favor of dexmedetomidine. The impact of marginal variable costs was given further consideration in another sensitivity analysis assessing only the estimated declining direct variable daily costs. Also, this analysis supported the same conclusions.

In terms of methodology, we considered a direct cost comparison based on consumed resources (cost-minimization) to be appropriate as dexmedetomidine has been shown to be non-inferior to standard care as measured by the proportion of time patients are maintained at the targeted, light to moderate sedation levels (Richmond Agitation and Sedation Scale: 0 to −3), without a difference in mortality [8]. The chosen approach should be considered to be conservative since it focuses purely on resource consumption and costs. We assumed that dexmedetomidine’s effectiveness, for which there is no simple definition, is as good as that of the standard sedatives. Any quality-of-life improvement that could potentially result from earlier extubation, patient’s improved ability to communicate with the nursing staff [6,8], or reduced neurocognitive adverse events [8] or delirium [6,28,29] were thus overlooked in this analysis.

An alternative methodological approach focusing on net costs could have been a cost-benefit analysis, in which not only the sedative consumption but also the established differences in the effectiveness of the treatment options are expressed in monetary terms and compared against each other resulting in ‘net monetary impact’ [12]. In this approach, resource utilization parameters, such as differences in time to extubation, duration of MV, and ICU length of stay, would have been considered to be constituents of benefit, to which eventually a monetary value would have been assigned. The result of such an analysis, ‘the net monetary benefit of dexmedetomidine’, would have been equal to the difference in the total ICU costs demonstrated in the present analysis, and thus the choice of analysis would not have impacted the conclusion.

We did not include any specific extra costs for adverse events. However, in a previous cost-minimization analysis of dexmedetomidine on the basis of the SEDCOM (Safety and Efficacy of Dexmedetomidine Compared to Midazolam) trial [6], such an approach was taken. In that analysis, even when the costs due to differences in adverse events were considered, dexmedetomidine was still associated with median total ICU cost savings of USD $9,679 compared with midazolam [30].

Since the scope of this analysis was economic, there are some distinct differences in the definitions and handling of the length of stay parameters, compared with the main clinical analysis [8]. In the present analysis, the intention was to reflect the total ICU costs per treatment group as realistically as possible, considering also the potential administrative delays, availability of a suitable ward bed, and so on, which finally determined when a patient actually got discharged. Therefore, the cost calculation was based on the observed actual length of ICU stay rather than the point ‘when medically fit for ICU discharge’ [8]. Also, for non-survivors, the length of ICU stay and the other relevant time points not yet reached were censored at time of death, or when moving to palliative care, as the accumulation of ICU costs ceased. For most patients who discontinued study medication prematurely, the actual times of all of the three relevant events had been captured as intended. In the relatively small number of cases (22 of a total of 990 patients) in which the subsequent data were lost to follow-up, the missing end points were worst ranked to the next actually recorded time point or (if none was available) to 45 days. The same imputation principle was applied on the two study withdrawals that took place early on, before MV or the ICU period had been completed. Thus, no significant bias should be expected from the way missing data were handled in this economic analysis. Nevertheless, since even non-significant imbalances between the treatment groups in censoring or imputation (or both) might introduce some degree of bias, we performed a sensitivity analysis including only those patients for whom observed data were available from all of the three ICU periods. Also, this analysis supported similar conclusions as the base case.

As a relatively large share of the total ICU costs may be of a fixed or at least semi-fixed nature [17,18], it may not be straightforward to realize the full savings potential indicated by our analysis, at least in the short term. However, even when only the direct variable ICU costs were included, the analysis still indicated savings with dexmedetomidine compared with standard care. Furthermore, from another perspective, even with the same staff costs, dexmedetomidine may potentially have implications for the cost-efficiency of the ICU, through freeing up beds faster. For example, if the ICU stay was reduced from 8.2 to 6.9 days (30 hours) as in the present case, then the same ICU staff could manage 19 additional patients per 100 patients sedated with dexmedetomidine, compared with standard sedatives. In ICU units with shorter underlying mean lengths of stay, the potential to increase the number of treated patients is even larger. Whether such increased patient turnover (and potentially a subsequently increased third-payer compensation to the hospital) is realistic depends on the organization and capacity of the referring wards and on the subsequent step-down units’ ability to admit the patients earlier.

The conclusion on the potential for cost savings with dexmedetomidine vs. standard care was robust in all sensitivity analyses performed. These included e.g. testing the impact of several variations of the ICU daily unit costs intended to represent different types of ICUs and economies (or countries), with higher or lower underlying costs vs. our base case, two scenarios considering the direct variable costs only, as well as a complementary cost analysis based on a well-established ICU resource use measure, the TISS points, collected daily in the PRODEX and MIDEX trials. In the analysis in which the share of direct variable costs was assumed to be 25% of the total ICU costs (which decline over time), the marginal direct cost estimate for the last ICU days was €347.50 (25% of €1,390 total cost of the Off MV days). This is in line with the $397 estimate reported by Kahn *et al*. [9] for the last ICU day and the $400 to $450 reported by Taheri *et al*. [11] for the marginal direct cost of the last hospital day. Even this conservative variable cost analysis still indicates that dexmedetomidine has the potential for leading to lower costs.

There was a statistically significant reduction in the duration of MV as well as in the cumulative sum of TISS points in comparison with midazolam or pooled standard care but not in comparison with propofol. Nevertheless, the likelihood of dexmedetomidine to result in lower total ICU costs exceeded 90% compared with pooled standard care and midazolam and was still relatively high (72%) even when compared with propofol. The conclusion of our analysis is also supported by other similar findings regarding lower resource use and ICU costs with dexmedetomidine versus both midazolam [30,31] and propofol [32].

These results reflect a rather heterogenic, general ICU patient population requiring prolonged sedation of over 24 hours. However, some relevant groups often requiring prolonged sedation, such as patients with acute severe neurological disorders, were excluded from the trials [8]. Furthermore, the reported cost savings were obtained within a rigorously controlled clinical trial implementing frequent sedation level control based on a validated sedation scale, daily sedation interruption, and spontaneous breathing trials. These interventions, recommended by recent guidelines, may help avoid unnecessarily deep sedation as such and are associated with improved outcomes, including reduced MV and ICU stay [33-36]. In practice, however, adherence to these best practices varies largely between hospitals [37-40]. Therefore, the cost consequences and impact of dexmedetomidine on ICU cost-efficiency in different patient groups in a real-life setting should be further assessed.

## Conclusions

This analysis demonstrates that, when targeting light to moderate sedation, dexmedetomidine represents an economically sound option that may provide ICU cost savings compared with the standard sedatives, primarily through reducing the time to extubation. If this can be translated to a shorter ICU stay, more patients can be managed with the same staff and fixed costs.

## Key messages

- This analysis indicates that, from an economic point of view, dexmedetomidine appears to be preferable option for light to moderate intensive care unit (ICU) sedation exceeding 24 hours.
- The potential of dexmedetomidine to achieve cost savings versus standard sedatives is primarily due to shorter time to extubation, which represents the most resource-intensive period in the ICU. To some extent, this benefit is also reflected in the total duration of mechanical ventilation and ICU stay but with higher uncertainty.
- From another perspective, these benefits may have implications on ICU cost-efficiency by freeing the ICU bed faster, allowing higher patient throughput with the same staff and fixed costs.

## Additional files

Additional file 1:
**List of the ethics committees that approved the clinical trial protocols.**
Additional file 2:
**Copy of the Therapeutic Intervention Scoring System-28 (TISS-28) scoring tool.**
Additional file 3: Table S1. Differences in study population and imputation approach in the current economic vs. the original clinical analysis with ITT data. Additional file 4: Table S2. Duration of the different intensive care unit (ICU) periods by mechanical ventilation use, and the duration of post-ICU ward stay. Additional file 5: Table S3. Acquisition costs of the study sedatives in pooled population and the MIDEX and PRODEX trials. Additional file 6: Figure S1. Combined bootstrapping analysis of the total intensive care unit (ICU) costs and the key underlying resource use parameter, time to extubation, in pooled population. Additional file 7: Table S4. Total intensive care unit (ICU) costs in patients with observed data available from all three time points, without any censoring or data imputations, in the pooled data analysis (S6). **Table S5.** Total intensive care unit (ICU) costs with dexmedetomidine and current standard care sedatives in the 18 centers whose routine daily ICU cost information the unit cost assumptions in this work were based on (S7). Additional file 8: Table S6. Details for Therapeutic Intervention Scoring System (TISS)-based intensive care unit (ICU) cost analyses including sedative costs-pooled data (S8a-b). Additional file 9: Table S7. Total intensive care unit (ICU) cost differences between the study treatment groups in the different sensitivity analyses (S1-S8), based on MIDEX. **Table S8.** Total ICU cost differences between the study treatment groups in the different sensitivity analyses (S1-S8), based on PRODEX.

## Abbreviations

ICU: intensive care unit
MV: mechanical ventilation
NIV: non-invasive ventilation
TISS: Therapeutic Intervention Scoring System
