# Supplementary material for: Dexmedetomidine versus standard care sedation with propofol or midazolam in intensive care: an economic evaluation
Source: Crit Care. 2015 Feb 19;19(1):67. doi: 10.1186/s13054-015-0787-y (PMC4391080; doi:10.1186/s13054-015-0787-y)
Supplement: Additional file 1: — List of the ethics committees that approved the clinical trial protocols. [file 13054_2015_787_MOESM1_ESM.pdf]

Additional File 1

List of the Ethics Committees that approved the clinical trial protocols

MIDEX STUDY

Belgium: Commissie Medische Ethiek (O.G.016) Reflectiegroep Biomedische Ethiek UZ Brussel; Comité d'éthique CHU Sart- Tilman, Liege; Ethische Commissie UZ Gent; Comité d'éthique UCL St. Luc, Bruxelles;

Estonia: Ethics Review of Tartu Office of Research and Institutional Development Tartu University Hospital

Finland: Ministry of Social Affairs and Health/ETENE

France: CPP Ile de France III Hôpital Tarnier-Cochin

Germany: Ethikkommission an der Medizinischen Fakultät Ernst-Moritz-Arndt-Universität Greifswald
Institut für Pharmakologie; Ethikkommission der medizinischen Fakultät, Tübingen; Ethikkommission an der Medizinischen Fakultät der Rheinischen Friedrich-Wilhelms-Universität Bonn

The Netherlands: METC Isala Klinieken Locatie Weezenlanden, Zwolle; Lokale Commissie Experimenteel Onderzoek op Mensen VieCuri Medisch Centrum voor Noord-Limburg; Lokale Toetsingscommissie Albert Schweitzer Ziekenhuis locatie Dordwijk; Medisch Ethische ToetsingsCommissie, St. Elisabeth Ziekenhuis; Toetsingscommissie Onderzoek Gelre Ziekenhuizenlocatie Lukas, Apeldoorn; Medisch Ethische Toetsingscommissie Amphia Ziekenhuis locatie Molengracht, Breda; Medisch Ethische Toetsingscommissie Kennemer Gasthuis; Medisch Ethische Toetsingscommissie VU medisch centrum, Amsterdam; Adviescommissie Mensgebonden Onderzoek UMC St. Raboud, Nijmegen

Norway: Regional Committee for Medical Research Ethics East Norway - (REK East) Pb

Switzerland: Kantonale Ethikkommission Bern; Kantonale Ethikkommission Universitätsspital Zürich; Kantonale Ethikkommission Spezialisierte Unterkommission Innere Medizin Universitätsspital Zürich

United Kingdom: REC - Cornwall and Plymouth REC and R&D - R&D Office, Derriford, Plymouth; REC - Solihull REC, Enfield, Redditch and R&D - R&D Directorate, Birmingham Heartlands Hospital, Birmingham; REC - South Birmingham REC - Enfield, Redditch and R&D - R&D Office, Queen Elizabeth Hospital, Edgbaston, Birmingham

PRODEX STUDY

Belgium: Ethisch Comité OL Vrouwziekenhuis Aalst; Ethische Commissies ZOL; Ethische Commissie UZ Gent

Finland: Ministry of Social Affairs and Health/ETENE

Germany: Ethikkommission des Fachbereichs Medizin; Ethikkommission der Medizinischen Fakultät Heidelberg Ethikkommission Erlangen Geschäftsstelle der Ethikkommission Medizinische Fakultät der FAU Erlangen-Nürnberg; Landesamt für Gesundheit und Soziales Geschäftsstelle der Ethikkommission des landes Berlin; Ethikkommission bei der Ärztekammer des Saarlandes; Ethikkommission an der Medizinischen Fakultät der Universität Leipzig; Geschäftsstelle der Ethikkommission der Medizinischen Fakultät der MLU Halle-Wittenberg; Ethikkommission der medizinischen Fakultät; Ethikkommission des Fachbereichs Medizin Der Johann Wolfgang Goethe-Universität

The Netherlands: Ethische Commissie, Westfriesgasthuis; Medisch-Ethische Toetsingscommissie Noord-Oost Brabant, Jeroen Bosch Ziekenhuis; TAC, Ziekenhuis Rivierenland Tiel; Medisch Ethische Toetsingscommissie, Amphia Ziekenhuis locatie Molengracht

Russia: Ethics Committee of State institution “B.V. Petrovsky, Russian Research Centre of Surgery of RAMS”, Moscow; Ethics Committee of Municipal healthcare institution “M.A. Podgorbunsky Municipal Clinical Hospital #3”; Ethics Committee at the Federal Control Service in Health Care & Social Development 4 bld.1, Moscow

Switzerland: Kantonale Ethikkommission Bern

The UK: REC - Wandsworth REC, South London REC Office (1), St Georges Hospital, and R&D - St George's Research Office, St George's University of London; REC - Suffolk LREC, West Suffolk Hospital, and R&D - R&D Office, West Suffolk Hospital; REC - Leeds West REC and R&D - R&D Directorate, General Infirmary, Leeds; REC - Lothian NHS Board, Deaconess House, Edinburgh, and R&D - Queens Medical Research Institute, Edinburgh
