# Supplementary material for: Dexmedetomidine versus standard care sedation with propofol or midazolam in intensive care: an economic evaluation
Source: Crit Care. 2015 Feb 19;19(1):67. doi: 10.1186/s13054-015-0787-y (PMC4391080; doi:10.1186/s13054-015-0787-y)
Supplement: Additional file 2: — Copy of the Therapeutic Intervention Scoring System-28 (TISS-28) scoring tool. [file 13054_2015_787_MOESM2_ESM.pdf]

Additional File 2: *Copy of the TISS-28-scoring tool*

TISS-28 (Therapeutic Intervention Scoring System-28)

Basic Activities	Points	
Standard monitoring. Hourly vital signs, regular registration and calculation of fluid balance.	5	yes no
Laboratory. Biochemical and microbiological investigations.	1	yes no
Single medication, any route (IV, PO, IM, etc.).	2	yes no
Multiple intravenous medications (more than 1 drug, single shots, or continuously)	3	yes
Routine dressing changes. Care and prevention of decubitus and daily dressing change.	1	yes no
Frequent dressing changes (at least one time per each nursing shift) and/or extensive wound care	1	yes no
Care of drains. All (except gastric tube).	3	yes no
**Cardiovascular Support**		
Single vasoactive medication. Any vasoactive drug.	3	yes no
Multiple vasoactive medications. More than1 vasoactive drug, disregard type and dose.	4	yes
Intravenous replacement of large fluid losses. Fluid replacement > 3 liters per square meter per day, disregard type of fluid administered.	4	yes no
Peripheral arterial catheter.	5	yes no
Left atrium monitoring. Pulmonary artery flotation catheter with or without cardiac output measurement.	8	yes no
Central venous line.	2	yes no
Cardiopulmonary resuscitation after arrest in the past 24 hours (single precordial percussion not included)	3	yes no
**Specific Interventions**		
Single specific interventions in the ICU. Naso or orotracheal intubation, introduction of a pacemaker, cardioversion, endoscopies, emergency surgery in the past 24 hours, gastric lavage. Routine interventions without consequences to the clinical condition of the patient, such as radiographs, echography, EKG, dressings or introduction of venous or arterial catheters, are not included.	3	yes no
Multiple specific interventions in the ICU. More than one, as described above.	5	yes
Specific interventions outside of ICU. Surgery or diagnostic procedures.	5	yes no

Ventilatory Support	Points	
Mechanical ventilation. Any form of mechanical or assisted ventilation with or without PEEP; with or without muscle relaxants; spontaneous breathing with PEEP).	5	yes no
Supplementary ventilatory support. Breathing spontaneously through endotracheal tube without PEEP; supplementary oxygen by any method except if mechanical ventilation parameters apply.	2	yes
Care of artificial airways. Endotracheal tube or tracheostoma.	1	yes no
Treatment for improving lung function. Thorax physiotherapy, incentive spirometry, inhalation therapy, intratracheal suctioning.	1	yes no
**Renal Support**		
Hemofiltration techniques. Dialytic techniques.	3	yes no
Quantitative urine output measurement.	2	yes no
Active diuresis (e.g. furosemid > 0.5 mg/kg/day for overload).	3	yes no
**Neurologic Support**		
Measurement of intracranial pressure.	4	yes no
**Metabolic Support**		
Treatment of complicated metabolic acidosis/alkalosis.	4	yes no
Intravenous hyperalimentation	3	yes no
Enteral feeding. Through gastric tube or other GI route (e.g. jejunostomy).	2	yes no

TISS-28 = 0

TISS-28 = SUM (points for activities performed) **Compute**

Clear

Time of nurse's care = 0

(One TISS-28 point equals 10.6 minutes of each 8 h nurse's shift)

TISS-76 correlation = 0

(Correlation beetwen TISS-28 and TISS-76: r = 0.93, r^2 = 0.86)
(TISS-28) = 3.33 + 0.97* (TISS-76)

Criteria of exclusion are applied in four conditions :

- "Multiple intravenous medications" excludes "Single medication";
- "Mechanical ventilation" excludes " Supplementary ventilatory support";
- "Multiple vasoactive medications" excludes "Single vasoactive medication";
- "Multiple specific interventions in the ICU" excludes "Single specific interventions in the ICU "

References

- Miranda DR et al. Simplified Therapeutic Intervention Scoring System : the TISS-28 items. Results from a multicenter study. *Crit Care Med.* 1996;24:64-73.
- Moreno R, Morais P. Validation of the simplified therapeutic intervention scoring system on an independent database. *Intensive Care Med.* 1997;23:640-644.
