# Supplementary material for: Dexmedetomidine versus standard care sedation with propofol or midazolam in intensive care: an economic evaluation
Source: Crit Care. 2015 Feb 19;19(1):67. doi: 10.1186/s13054-015-0787-y (PMC4391080; doi:10.1186/s13054-015-0787-y)
Supplement: Additional file 3: Table S1. — Differences in study population and imputation approach in the current economic vs. the original clinical analysis with ITT data. [file 13054_2015_787_MOESM3_ESM.pdf]

Table S1. Differences in study population and imputation approach in the current economic vs. the original clinical analysis with ITT data [8]. The number of patients excluded from the economic analysis and the number of patients affected by the revised application of data imputations regarding ICU stay are presented. The number of the relevant patients by study and treatment group are shown; D = dexmedetomidine, M = midazolam, P = propofol, SC= standard care i.e. midazolam and propofol pooled 1:1.

Group	Approach in the present economic analysis	Approach in the original clinical analysis	MIDEX		PRODEX		Pooled data	
			D	M	D	P	D	SC
**Patients who never received any study drug, or withdrew their consent**	Excluded from the entire economic analysis	Included in analysis	2	1	5	0	7	1
**Patients who died in ICU (i.e. no date of discharge)**	Data censored to the time of death	Imputation to 45 days	44	38	27	31	71	69
**Patients discharged to palliative care**	Data censored to the time of moving out of the ICU	Imputation to 45 days	1	0	0	1	1	1

Missing end points not yet reached were anchored to the time of death or moving to palliative care in the case of 72 and 70 **non-survivors** (pooled data) in the dexmedetomidine and standard care groups, respectively. Within the patient group who **discontinued study medication prematurely** (231), most end point data had been captured to 45 days, as intended by the protocol; worst ranking imputation was applied on the missing end points in 22 of these patients, if calculating any end point data lost. As for **the patients who withdrew from the entire study**, for 26 of 28 such patients, all three relevant end points up to ICU discharge had been actually recorded before the study withdrawal took place. In case of two early study withdrawals - one patient without any relevant data, the other with only extubation time known - the remaining, later end points for which the data were lost, were worst ranked to 45 days as also done in the original clinical analysis.

.
