# Supplementary material for: Dexmedetomidine versus standard care sedation with propofol or midazolam in intensive care: an economic evaluation
Source: Crit Care. 2015 Feb 19;19(1):67. doi: 10.1186/s13054-015-0787-y (PMC4391080; doi:10.1186/s13054-015-0787-y)
Supplement: Additional file 4: Table S2. — Duration of the different intensive care unit (ICU) periods by mechanical ventilation use, and the duration of post-ICU ward stay. [file 13054_2015_787_MOESM4_ESM.pdf]

Table S2. Duration of the different ICU periods by mechanical ventilation needs, and duration of post-ICU ward stay. The data are expressed as mean (standard deviation [SD]) and median (interquartile range [IQR]) number of days (24 hr periods) by treatment group. Time at ward was calculated until discharge or death, up to the end of the follow-up period of 45 days. Possible readmission is not included, missing discharge dates for reasons other than death were imputed to day 45.

	**Mean (SD)**		**Median (IQR)**	
	**dexmedetomidine**	**pooled standard care**	**dexmedetomidine**	**pooled standard care**
**Days in ICU on invasive MV**	6.9 (9.1)	8.1 (9.2)	3.7 (1.9 - 7.8)	4.9 (2.2 - 9.6)
**Days in ICU on NIV**	1.1 (4.4)	0.8 (3.0)	0.0 (0.0 - 0.0)	0.0 (0.0 - 0.0)
**Days in ICU off MV**	3.3 (5.7)	3.5 (5.5)	1.6 (0.3 - 3.2)	1.8 (0.6 - 3.9)
**Days at ward**	11.1 (11.7)	10.9 (10.7)	8.0 (1.0 - 16.3)	8.2 (1.1 - 16.9)
	**dexmedetomidine**	**midazolam**	**dexmedetomidine**	**midazolam**
**Days in ICU on invasive MV**	7.7 (9.4)	9.0 (9.6)	4.0 (2.5 - 8.8)	6.0 (3.1 - 6.0)
**Days in ICU on NIV**	0.6 (3.3)	0.7 (3.6)	0.0 (0.0 - 0.0)	0.0 (0.0 - 0.0)
**Days in ICU off MV**	3.4 (5.9)	3.6 (5.3)	1.9 (0.3 - 3.3)	2.1 (1.0 - 3.9)
**Days at ward**	10.7 (12.0)	10.4 (10.2)	6.8 (0.8 - 17.0)	8.1 (1.0 - 16.1)
	**dexmedetomidine**	**propofol**	**dexmedetomidine**	**propofol**
**Days in ICU on invasive MV**	6.2 (8.8)	7.3 (8.8)	2.9 (1.6 - 6.8)	3.8 (1.8 - 9.6)
**Days in ICU on NIV**	1.6 (4.9)	0.8 (2.4)	0.0 (0.0 - 0.0)	0.0 (0.0 - 0.2)
**Days in ICU off MV**	3.1 (5.5)	3.3 (5.6)	1.4 (0.3 - 3.2)	1.1 (0.3 - 3.8)
**Days at ward**	11.5 (11.3)	11.5 (11.1)	9.1 (3.0 - 16.3)	8.9 (1.3 - 17.1)
