# Supplementary material for: Dexmedetomidine versus standard care sedation with propofol or midazolam in intensive care: an economic evaluation
Source: Crit Care. 2015 Feb 19;19(1):67. doi: 10.1186/s13054-015-0787-y (PMC4391080; doi:10.1186/s13054-015-0787-y)
Supplement: Additional file 5: Table S3. — Acquisition costs of the study sedatives in pooled population and the MIDEX and PRODEX trials. [file 13054_2015_787_MOESM5_ESM.pdf]

Table S3. Acquisition costs of the study sedatives in pooled population and the MIDEX and PRODEX trials, expressed as €/patient.

	**Mean (SD)**		**Difference in means, €**	**Median (IQR)**		**Difference in medians, €**
	**dexmede-tomidine**	**standard care**		**dexmede-tomidine**	**standard care**	
**Pooled**	313.7 (372.1)	104.6 (126.6)	**209.1**	187.5 (80.7 - 388.0)	59.0 (25.8 - 138.4)	**128.5**
**MIDEX**	270.5 (382.0)	68.7 (77.2)	**201.8**	140.8 (53.9 - 302.3)	36.0 (16.2 - 99.0)	**104.8**
**PRODEX**	357.0 (357.4)	140.9 (153.8)	**116.1**	237.5 (120.6 - 471.6)	89.7 (39.8 - 181.3)	**147.8**

In terms of quantity, mean total dexmedetomidine consumption was 15 x 200 mcg ampoules (mean 3.0 mg; median 1.6 mg) in MIDEX, while 86 x 5 mg of midazolam (mean 429.6 mg, median 224.9 mg) was used. Mean duration of dexmedetomidine use was 2.53 days (60.6 hours), and of midazolam 3.25 days (78 hours). In PRODEX mean total dexmedetomidine consumption was 20 x 200 mcg (4.0 mg, median 2.6 mg) and propofol consumption 12 x 1,000 mg (mean 11 745.2 mg, median 7,473.8 mg). Mean duration of dexmedetomidine use was 2.51 days (60.3 hours) and of propofol 3.37 days (80.8 hours).
