# Supplementary material for: Dexmedetomidine versus standard care sedation with propofol or midazolam in intensive care: an economic evaluation
Source: Crit Care. 2015 Feb 19;19(1):67. doi: 10.1186/s13054-015-0787-y (PMC4391080; doi:10.1186/s13054-015-0787-y)
Supplement: Additional file 6: Figure S1. — Combined bootstrapping analysis of the total intensive care unit (ICU) costs and the key underlying resource use parameter, time to extubation, in pooled population. [file 13054_2015_787_MOESM6_ESM.pdf]

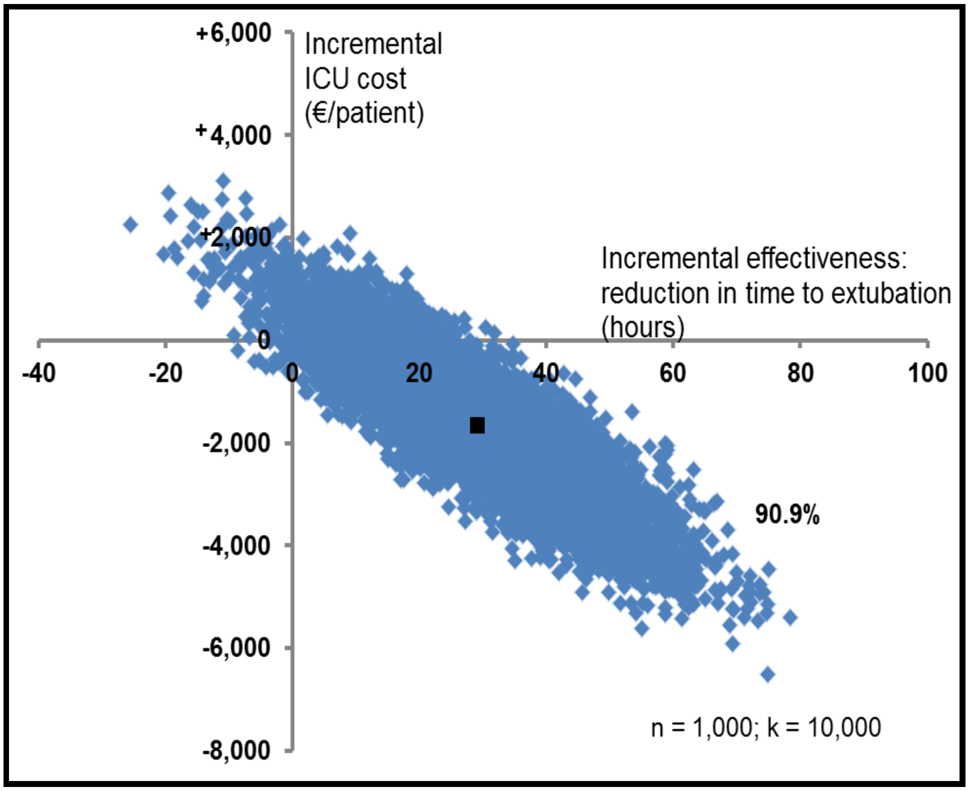

Figure S1. Combined bootstrapping analysis of the total ICU costs and the key underlying resource use parameter, time to extubation, in pooled population. This analysis shows that there is a 90.9% likelihood of dexmedetomidine to shorten the time to extubation and simultaneously reduce total ICU costs, compared to standard sedatives. The average difference between the treatments (marked by the black square) in median time to extubation was 29 hours and in median total ICU costs €1,656, both lower with dexmedetomidine. (n= total number of patients sampled per round; k= number of repetitions of the sampling in the bootstrapping).
