# Supplementary material for: Dexmedetomidine versus standard care sedation with propofol or midazolam in intensive care: an economic evaluation
Source: Crit Care. 2015 Feb 19;19(1):67. doi: 10.1186/s13054-015-0787-y (PMC4391080; doi:10.1186/s13054-015-0787-y)
Supplement: Additional file 7: Table S4. — Total intensive care unit (ICU) costs in patients with observed data available from all three time points, without any censoring or data imputations, in the pooled data analysis (S6). Table S5. Total intensive care unit (ICU) costs with dexmedetomidine and current standard care sedatives in the 18 centers whose routine daily ICU cost information the unit cost assumptions in this work were based on (S7). [file 13054_2015_787_MOESM7_ESM.pdf]

Table S4. Total ICU costs in patients with observed data available from all three time points, without any censoring or data imputations, in the pooled data analysis (S5).

**Mean (SD)**		**Difference in means, €**	**Median (IQR)**		**Difference in medians, €**
**dexmedetomidine** (n = 366)	**standard care** (n = 361)		**dexmedetomidine** (n = 366)	**standard care** (n = 361)	
14,983 (12,782)	18,196 (15,349)	**-3,213**	10,362 (6,736-8,954)	12,284 (7,557-23,821)	**-2,462**

Table S5. Total ICU costs with dexmedetomidine and current standard care sedatives in the 18 centers whose routine daily ICU cost information the unit cost assumptions in this work were based on (S6). These 18 study sites included a total of 339 patients in the 2 trials (34% of pooled total N). 36% of these participated in the MIDEX trial, 64% in the PRODEX trial. Most of these patients were treated at trial centers in Finland and Switzerland (195 and 66, respectively).

**Mean (SD)**		**Difference in means, €**	**Median (IQR)**		**Difference in medians, €**
**dexmedetomidine** (n = 171)	**standard care** (n = 168)		**dexmedetomidine** (n = 171)	**standard care** (n = 168)	
17,076 (18,003)	18,869 (16,622)	**-1,793**	9,588 (6,539 - 20,702)	14,125 (7,468 - 24,672)	**-4,567**
