# Supplementary material for: Dexmedetomidine versus standard care sedation with propofol or midazolam in intensive care: an economic evaluation
Source: Crit Care. 2015 Feb 19;19(1):67. doi: 10.1186/s13054-015-0787-y (PMC4391080; doi:10.1186/s13054-015-0787-y)
Supplement: Additional file 8: Table S6. — Details for Therapeutic Intervention Scoring System (TISS)-based intensive care unit (ICU) cost analyses including sedative costs-pooled data (S8a-b). [file 13054_2015_787_MOESM8_ESM.pdf]

Table S6. Details for TISS-based ICU cost analyses in pooled data (S7a-b).

Unit cost assumptions of a) €40 (top row) and b) €50 per TISS point (bottom row) were assumed in this analysis.

**Mean (SD)**		**Difference in means, €**	**Median (IQR)**		**Difference in medians, €**
**dexmedetomidine** (n = 487)	**standard care** (n = 489)		**dexmedetomidine** (n = 487)	**standard care** (n = 489)	
14,713 (15,136)	16,212 (14,454)	**-1,499**	9,869 (6,260 - 17,202)	11,317 (7,100 - 20,286)	**-1,448**
18,313 (18,890)	20,239 (18,056)	**-1,926**	12,309 (7,796 - 21,478)	14,092 (8,867- 25,350)	**-1,782**
