# Supplementary material for: Dexmedetomidine versus standard care sedation with propofol or midazolam in intensive care: an economic evaluation
Source: Crit Care. 2015 Feb 19;19(1):67. doi: 10.1186/s13054-015-0787-y (PMC4391080; doi:10.1186/s13054-015-0787-y)
Supplement: Additional file 9: Table S7. — Total intensive care unit (ICU) cost differences between the study treatment groups in the different sensitivity analyses (S1-S8), based on MIDEX. Table S8. Total ICU cost differences between the study treatment groups in the different sensitivity analyses (S1-S8), based on PRODEX. [file 13054_2015_787_MOESM9_ESM.pdf]

Table S7. Total ICU cost differences between the study treatment groups in the different sensitivity analyses (S1-S8), based on MIDEX

Sensitivity analysis of MIDEX: dexmedetomidine vs. midazolam	Difference in mean costs, €	Difference in median costs, €
S1. Standard sedative set to cost zero	**- 2,467**	**- 3,466**
S2. Dexmedetomidine cost increased to €22 per 200 mcg (€0.11 per 1 mcg)	**- 2,476**	**- 3,557**
S3. Cost of a NIV day (24 hr) decreased to € 1,390	**- 2,519**	**- 3,557**
S4. Cost of a NIV day (24 hr) increased to €1,850	**- 2,541**	**- 3,573**
S5. Patients with observed data available from all three time periods (without any censoring or imputations)	**- 3,900**	**- 3,675**
S6. MIDEX patients of those 18 study centers, from which routine ICU costs were available. N (dexmedetomidine):63; propofol: 58	**- 907**	**- 3,351**
S7a. TISS-based total ICU costs, at unit cost €40 per TISS point	**- 2,444**	**- 2,364**
S7b. TISS-based total ICU costs, at unit cost of €50 per TISS point	**-3,105**	**-2,972**
S8a. Only declining, direct variable daily costs included, assuming they represent 25% of the total ICU costs	**-483**	**-704**
S8b. Only the periodically declining, direct variable costs considered, assuming they represent 50% of the total ICU costs	**-1,167**	**-1,772**

NIV= non-invasive ventilation, TISS= Therapeutic Intervention Scoring System

Table S8. Total ICU cost differences between the study treatment groups in the different sensitivity analyses (S1-S8), based on PRODEX

**Sensitivity analysis of PRODEX: dexmedetomidine vs. propofol**	**Difference in mean costs, €**	**Difference in median costs, €**
S1. Standard sedative set to cost zero	**- 606**	**- 1,265**
S2. Dexmedetomidine cost €22 per 200 mcg (€0.11 per 1 mcg)	**- 668**	**- 1,234**
S3. Cost of a NIV day decreased to €1,390 per 24 hours	**- 1,022**	**- 1,266**
S4. Cost of a NIV day increased to €1,850 per 24 hours	**- 1,614**	**- 2,552**
S5. Patients with observed data available from all three time periods (without any censoring or imputations)	**- 2,486**	**- 1,182**
S6. PRODEX patients of those 18 study centers, from which routine ICU costs were available. N (dexmedetomidine):108; propofol: 110	**- 2,571**	**- 2,207**
S7a. TISS-based total ICU costs, at unit cost of €40 per TISS point	**- 517**	**- 781**
S7b. TISS-based total ICU costs, at unit cost of €50 per TISS point	**-700**	**-1,089**
S8a. Only the declining, direct variable daily costs included, assuming they represent 25% of the total ICU costs	**-25**	**-660**
S8b. Only declining, direct variable daily costs included, assuming they represent 50% of the total ICU costs	**-266**	**-1,550**

NIV= non-invasive ventilation, TISS= Therapeutic Intervention Scoring System
